# TRIM71 suppresses tumorigenesis via modulation of Lin28B-let-7-HMGA2 signaling

**DOI:** 10.18632/oncotarget.13036

**Published:** 2016-11-03

**Authors:** Jinlong Yin, Tae-Hoon Kim, Nayun Park, Daye Shin, Hae In Choi, Sungchan Cho, Jong Bae Park, Jong Heon Kim

**Affiliations:** ^1^ Department of System Cancer Science, Graduate School of Cancer Science and Policy, National Cancer Center, Goyang, Gyeonggi, Korea; ^2^ Cancer Cell and Molecular Biology Branch, Research Institute, National Cancer Center, Goyang, Gyeonggi, Korea; ^3^ Specific Organs Cancer Branch, Research Institute, National Cancer Center, Goyang, Gyeonggi, Korea; ^4^ Anticancer Agent Research Center, Korea Research Institute of Bioscience & Biotechnology, Ochang, Korea

**Keywords:** TRIM71, Lin28B, let-7, HMGA2, tumorigenesis

## Abstract

TRIM71 (tripartite motif-containing 71) belongs to the TRIM-NHL protein family, which plays a conserved role in regulating early development and differentiation. However, the molecular functions of TRIM71 have remained largely unknown. Here, we explored the role of TRIM71 together with modulation of Lin28B-let-7-HMGA2 (high-mobility group AT-hook 2) signaling in tumorigenesis. TRIM71 overexpression opposed Lin28B-induced transformation in primary cells and inhibited tumor formation in a mouse model. Specific knockdown of TRIM71 expression increased cancer cell proliferation and invasion. Conversely, overexpression of wild-type TRIM71 in non-small cell lung carcinoma (NSCLC) cells in which Lin28B-let-7-HMGA2 signaling was conserved decreased both cancer cell phenotypes. More importantly, overexpression of an ubiquitin transfer activity-deficient TRIM71 mutant in NSCLC cells had no effect on proliferation or invasion, regardless of the conservation status of Lin28B-let-7-HMGA2 signaling. The tumorigenic inhibitory action of TRIM71 was antagonized by overexpression of the TRIM71 downstream targets, Lin28B and HMGA2. Furthermore, a bioinformatics analysis revealed that TRIM71 expression was downregulated in various types of cancer tissue from patients. Taken together, these data indicate that TRIM71 acts through post-transcriptional repression of Lin28B and subsequent modulation of let-7-HMGA2 signaling during tumorigenesis to potentially function as a tumor suppressor.

## INTRODUCTION

TRIM71 (tripartite motif-containing 71), a member of the TRIM-NHL family consisting of TRIM2, -3, -32, and -71, was initially discovered as a temporal cell fate regulator in *Caenorhabditis elegans* early development and has since been shown to be relatively conserved in metazoans. It is also known as *lin-41* (lineage variant 41), which is a genetic suppressor of a *let-7* loss-of-function mutant [1, 2].

TRIM71 shares structural similarities in the N-terminal tripartite motif (TRIM), composed of a RING domain, B-box and coiled-coil regions, with other TRIM-domain–containing protein family members. Like other TRIM-NHL proteins, TRIM71 also has unique C-terminal features, including a filamin domain and an NHL (NCL-1, HT2A2, and LIN-41)-repeat motif. All members of the TRIM-NHL family possess functional E3 ubiquitin ligase activity, which is critically dependent on the RING (Really Interesting New Gene) domain in the N-terminus [1–3]. With the exception of this RING domain, the physiological functions of other structurally defined motifs in TRIM71 remain unknown.

Several studies have reported that the RING motif of TRIM71 is essential for ubiquitin transfer and subsequent target protein degradation or stabilization. TRIM71 acts as a specific E3 ubiquitin ligase for the RISC (RNA-induced silencing complex) catalytic component, Ago2 (argonaute 2), which is essential for microRNA biogenesis and targeting [4]. Moreover, the mouse form of TRIM71 (mLin41) has been shown to stabilize SHCBP1 (Shc SH2-binding protein 1), an important component of fibroblast growth factor (FGF) signaling, and enhance FGF signaling in neuronal progenitor cells [5].

The RNA-binding protein Lin28B, an important substrate of TRIM71-mediated ubiquitination, negatively regulates the biogenesis of the tumor-suppressive let-7 family at the post-transcriptional level [6]. Lin28B, as well as it paralog Lin28A, specifically interacts with the loop sequence of pre-let-7 microRNA and mediates terminal oligo-uridylation and induces destabilization of the precursor [7]. Negative modulation of let-7 microRNAs by Lin28B suppresses HMGA2 (high mobility group AT-hook 2), Ras, and Myc - oncogenic downstream targets of let-7 [8–11].

Notably, Lin28B overexpression is frequently observed in various cancers, such as hepatocellular carcinoma, colorectal cancer, pancreatic cancer and non-small cell lung carcinoma (NSCLC), and is associated with induction of neuroblastoma [12–18]. Moreover, ectopic expression of Lin28B in NIH/3T3 cells stimulates cellular transformation, possibly through repression of let-7 microRNA expression [18]. Therefore, Lin28B, acting as a post-transcriptional modulator, is usually considered to possess oncogenic properties.

Our previous report demonstrated that human Lin28B activity is negatively regulated at the protein level by ubiquitin-dependent proteasomal degradation mediated by TRIM71. Specific inhibition of Lin28B by TRIM71 subsequently modulates let-7 microRNA, a specific Lin28B cellular target, and represses HMGA2 protein translation [6].

On the basis of this critical observation and various previous reports, we investigated the potential role of TRIM71 in tumorigenesis. Intriguingly, TRIM71 suppressed tumorigenesis in a manner that dependent on its cellular ubiquitination target Lin28B. Moreover, subsequent modulation of let-7 and its post-transcriptional target HMGA2 were essential for the anti-tumorigenic action of TRIM71.

## RESULTS

### TRIM71 suppresses the cellular-transforming activity of Lin28B

**Figure 1:**
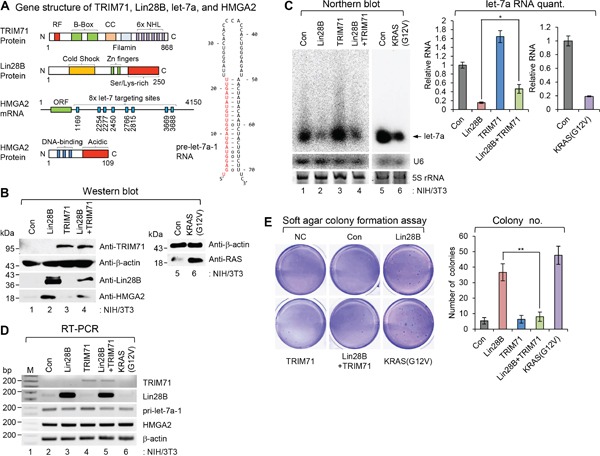
TRIM71 suppresses the cellular-transforming activity of Lin28B **A.** Gene structures of TRIM71, Lin28B, pre-let-7a-1, and HMGA2. Numbers within images represent amino acid or nucleotide position of each gene. Red colored nucleotides in the pre-let-7a-1 RNA represent mature let-7a sequence. RF, RING finger motif; CC, coiled-coil domain; ORF, open reading frame. **B-D.** Overexpression of Lin28B promotes cellular transformation in NIH/3T3 cells. However the transformation potential of Lin28B was abrogated by TRIM71 co-expression. NIH/3T3 cells infected with pMSCV-neo/pBABE-puro, pMSCV-neo/pBABE-puro-Lin28B, pMSCV-neo-TRIM71/pBABE-puro, pMSCV-neo-TRIM71/pBABE-puro-Lin28B, and pMSCV-neo/pBABE-puro-KRAS(G12V) and selected with puromycin and G418 together. Relative expression of each protein and RNA species determined by western blot (WB) (B), northern blot (NB) (C) [SD is indicated in the graph (*p<0.05). The *p*-value compares the Lin28B to Lin28B+TRIM71], and semi-quantitative RT-PCR (RT-PCR) (D). **E.** Soft agar colony formation assays were performed as described in MATERIALS AND METHODS. 5,000 cells/well of virus infected stable NIH/3T3 cells were plated on 6-well plates for soft agar colony formation assay. Colonies bigger than 50 μm were counted after 4 weeks. Colonies were counted from three wells and the average numbers are represented in graph. Data represent the mean values of at least three independent experiments performed in triplicate (**p<0.01). Error bars in the graph represent ± SD, and the *p*-value compares the Lin28B to Lin28B+TRIM71. NC; negative control without cell plating. Con; pMSCV-neo/pBABE-puro.

As depicted in Figure 1A and demonstrated by our previous report, TRIM71 contains a specific RING finger motif in its N-terminal region that mediates ubiquitin transfer to the Lin28B. The specific E3 ubiquitin ligase activity of TRIM71 negatively regulates Lin28B protein levels post-transcriptionally. Notably, this region is also critical for protein-protein interactions with Lin28B [6].

As demonstrated previously, the C-terminal region of Lin28B, which contains a number of lysine and serine residues, is critical for interactions with TRIM71. This region is also critically important in discriminating between Lin28B and its paralog Lin28A [6, 13, 19]. Both Lin28B and Lin28A specifically interact with the loop sequence of pre-let-7, an interaction that modulates oligo-uridylation and degradation of pre-let-7; the maturation process leads to the well-known post-transcriptional tumor suppressor, let-7 [7]. An essential downstream target of let-7 is HMGA2 mRNA, which contains eight let-7 target sites within its 3′-untranslated region (3′-UTR) [9, 10]. This post-transcriptional modulation of Lin28B-let-7-HMGA2 signaling motivated us to investigate whether TRIM71 has a potential role in this signaling during tumorigenesis.

Lin28B overexpression in NIH/3T3 cells leads to significant depletion of let-7 and a concomitant increase in the abundance of let-7 target proteins. This negative regulation of let-7 microRNA by Lin28B activates oncogenic signaling and induces cellular transformation of NIH/3T3 cells [18]. Our previous report clearly demonstrated TRIM71-mediated poly-ubiquitination and degradation of Lin28B protein in cells [6]. Therefore, we first examined whether the cellular transformation initiated by Lin28B is suppressed by TRIM71.

As shown in Figure 1B and 1C, overexpression of Lin28B in NIH/3T3 cells via retroviral transduction significantly depleted let-7a, leading to upregulation of the let-7a target protein, HMGA2. Semi-quantitative reverse transcription-polymerase chain reaction (RT-PCR) analyses (Figure 1D) showed that negative regulation of let-7a microRNA and positive regulation of HMGA2 protein did not reflect transcriptional modulation of either gene transcript. Notably, these changes in let-7a microRNA and HMGA2 protein were partially abrogated by co-transduction of Lin28B- and TRIM71-expressing retroviruses.

As shown in Figure 1B, co-expression of TRIM71 and Lin28B caused a reduction in Lin28B protein level in NIH/3T3 cells, consequently increasing the level of let-7a RNA and repressing HMGA2 protein - results opposite those obtained following overexpression of Lin28B alone. NIH/3T3 cells overexpressing Lin28B or TRIM71 alone showed let-7a RNA and HMGA expression patterns similar to those previously reported by Lee et al [6] in 293T cells. However, co-expression of Lin28B and TRIM71 did not completely restore let-7 levels, possibly owing to an inhibitory effect of residual Lin28B on pre-let-7 [6].

As shown in Figure 1E, NIH/3T3 cells overexpressing Lin28B formed colonies in soft agar at a frequency comparable to that of cells expressing mutant KRAS [KRAS(G12V)]. Expression of Lin28B resulted in an increase in both colony size (data not shown) and number compared with control NIH/3T3 cell lines and those expressing TRIM71 alone. NIH/3T3 cells co-expressing Lin28B and TRIM71 displayed reduced colony size and number, similar to control NIH/3T3 cell lines and those expressing TRIM71 alone.

These *in vitro* results, taken together with the inhibitory effect of TRIM71 on cellular-transformation potential, suggest that negative modulation of Lin28B-let-7-HMGA2 signaling by TRIM71 is important for the cellular transformation process.

### TRIM71 inhibits tumor growth initiated by Lin28B

**Figure 2:**
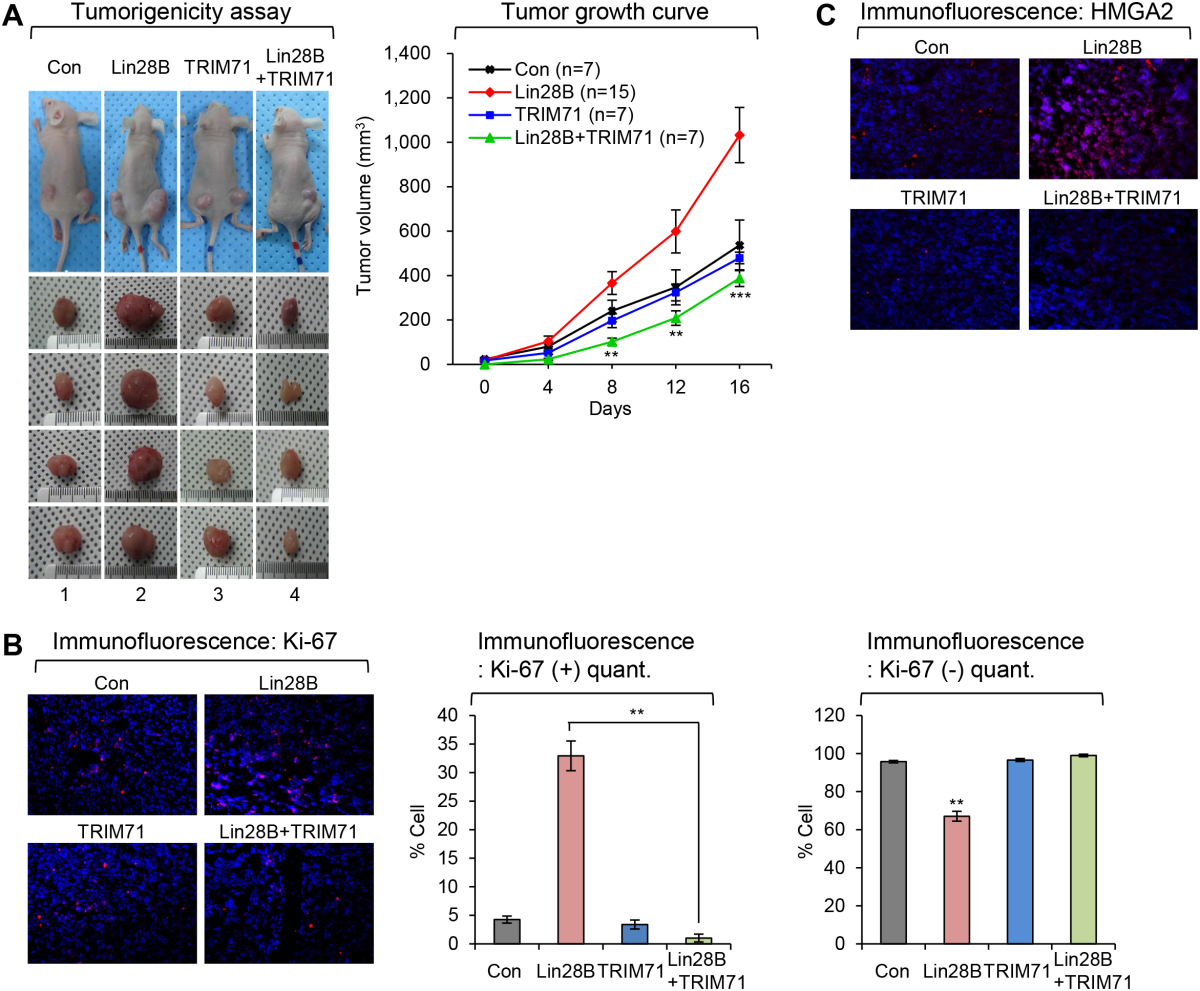
TRIM71 inhibits tumor growth initiated by Lin28B **A.** Stable virus infected NIH/3T3 cell lines which expressing Lin28B, TRIM71, Lin28B+TRIM71 and KRAS(G12V) were injected subcutaneously into the hip area on both sides of nude mice. Stable virus infected NIH/3T3 cells were propagated *in vivo* for five weeks. Tumors were measured from a point of growing up to be counted as 0 day. KRAS(G12V) is positive control for tumor formation in mice. The tumor volume of KRAS(G12V) has grown to over 1,000 mm^3^ rapidly until for 14 days (data not shown). SDs are indicated in the graph (**p<0.01 and ***p<0.001). The *p*-value compares the Lin28B to Lin28B+TRIM71. **B.** Ki-67 staining in tumors derived from the subcutaneously injected with stable virus infected NIH/3T3 cells which expressing control, Lin28B, TRIM71, and Lin28B+TRIM71. Representative photos (left) and quantitative data (right) were shown. **p<0.01. **C.** HMGA2 staining in tumors derived from the subcutaneously injected with stable virus infected NIH/3T3 cells which expressing control, Lin28B, TRIM71, and Lin28B+TRIM71.

To establish a functional role of TRIM71 in Lin28B-mediated cellular transformation and tumor formation, we directly injected NIH/3T3 cells used in soft agar colony-formation assay into nude mice and compared their abilities to form tumors (Figure 2). Overexpression of Lin28B alone significantly enhanced tumor formation, whereas co-expression of Lin28B and TRIM71 markedly inhibited tumor growth (Figure 2A). For quantitative comparison, we repeated the same experiment with 25 additional mice (initial numbers of animal are 15). An analysis of combined results from all 36 mice revealed that the volumes of tumors formed by Lin28B-overexpressing cells were significantly larger than those formed by cells co-expressing Lin28B and TRIM71. Staining for the proliferation marker Ki-67 confirmed that cell proliferation was increased in Lin28B-overexpressing tumors compared with those from control mice; however, co-expression of Lin28B and TRIM71 suppressed Lin28B-induced cell proliferation (Figure 2B). We also monitored the expression of the Lin28B downstream target HMGA2 by immunofluorescence staining (Figure 2C). These analyses showed that Lin28B overexpression significantly increased HMGA2 expression, an effect that was attenuated by co-expression of TRIM71 (Figure 2C). These results suggest that TRIM71 inhibits tumor formation by modulating Lin28B-let-7-HMGA2 signaling *in vivo*.

### Depletion of TRIM71 promotes proliferation and invasion of colorectal carcinoma Caco-2 and human embryonal carcinoma Tera-1 cells

As shown in Figures 1 and 2, TRIM71 suppressed cellular transformation and tumor formation by directly inhibiting the activity of the oncoprotein, Lin28B. Next, we sought to extend this analysis beyond non-transformed and immortalized primary cells, such as NIH/3T3, by investigating similar physiological phenomena in colorectal carcinoma Caco-2 and human embryonal carcinoma Tera-1 cell lines.

**Figure 3:**
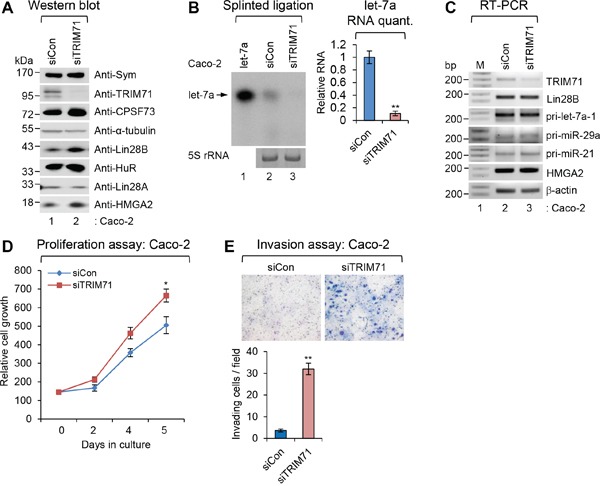
Depletion of TRIM71 promotes proliferation and invasion of colorectal carcinoma Caco-2 cells **A.** The level of symplekin, TRIM71, CPSF73, α-tubulin, Lin28B, HuR, Lin28A, and HMGA2 proteins were confirmed by WB after specific knockdown of TRIM71 in colorectal carcinoma Caco-2 cells. **B.** Splinted ligation was performed with [^32^P] 5′-end-labeled oligonucleotide probe specific for mature let-7a as described in MATERIALS AND METHODS. Aliquot of total RNAs were resolved in 12% SequaGel together as the loading control (5S rRNA). The experiments were repeated at least three times with similar results. The images shown in panels are representative one. **p<0.01. **C.** The level of TRIM71, Lin28B, HMGA2, β-actin mRNAs, and various pri-miRNAs were analyzed with RT-PCR. **D.** Proliferation assay was performed in Caco-2 cells transfected with scrambled (siCon) or TRIM71 (siTRIM71) specific siRNA. *p<0.05 **E.** Invasion assay was performed in Caco-2 cells transfected with scrambled (siCon) or TRIM71 (siTRIM71) specific siRNA. Representative photos (top) and quantitative data of invaded cells (lower) are shown. **p<0.01.

First, we tested whether the TRIM71-Lin28B-let-7-HMGA2 signaling pathway operated in those cancer cell lines. As expected, small interfering RNA (siRNA)-mediated depletion of endogenous TRIM71 induced the accumulation of endogenous Lin28B protein in both cell lines (Figure 3A and Supplementary Figure S1A), but did not affect expression of Lin28A [19]. Based on these findings, we expected that increased Lin28B would inhibit maturation of let-7. Consistent with this prediction, an examination of let-7 microRNA levels after TRIM71 knockdown showed that mature let-7a was completely depleted in Caco-2 (Figure 3B) and Tera-1 (Supplementary Figure S1B) cells. A previous report on Huh-7, P19, and NCCIT cell lines, in which TRIM71-Lin28B-let-7-HMGA2 signaling is conserved, showed similar results [6]. To exclude the possibility that primary let-7a-1 (pri-let-7a-1) is transcriptionally regulated by TRIM71 knockdown, we performed RT-PCR using primers specific for several pri-miRNAs. Knockdown of TRIM71 did not affect pri-let-7a-1 levels or those of other pri-miRNAs; Lin28B and HMGA2 mRNAs were similarly unaffected (Figure 3C and Supplementary Figure S1C).

Since TRIM71 inhibits tumor growth initiated by Lin28B, we examined the function of TRIM71 in suppressing tumorigenic phenotypes. siRNA-mediated TRIM71 knockdown increased the proliferation of both Caco-2 and Tera-1 human cancer cell lines compared with control siRNA-transfected cells (Figure 3D and Supplementary Figure S1D). Invasion assays revealed that TRIM71 knockdown also increased cell invasion compared with control cells (Figure 3E and Supplementary Figure S1E), suggesting that TRIM71 is required for tumor invasion. These results collectively indicate that TRIM71 is a critical regulator of tumorigenesis in cancers in which Lin28B-let-7-HMGA2 signaling is present.

### Overexpression of wild-type TRIM71, but not its RING finger mutant, abrogates proliferation and invasion of NCI-H1299 NSCLC cells

In our previous report, we suggested that the N-terminal RING finger motif of TRIM71 is essential for ubiquitination activity, interaction with Lin28B, and let-7 biogenesis [6]. Therefore, we hypothesized that this motif also negatively influences general tumorigenic phenotypes (tumor invasion and proliferation) via Lin28B-let-7-HMGA2 signaling.

**Figure 4:**
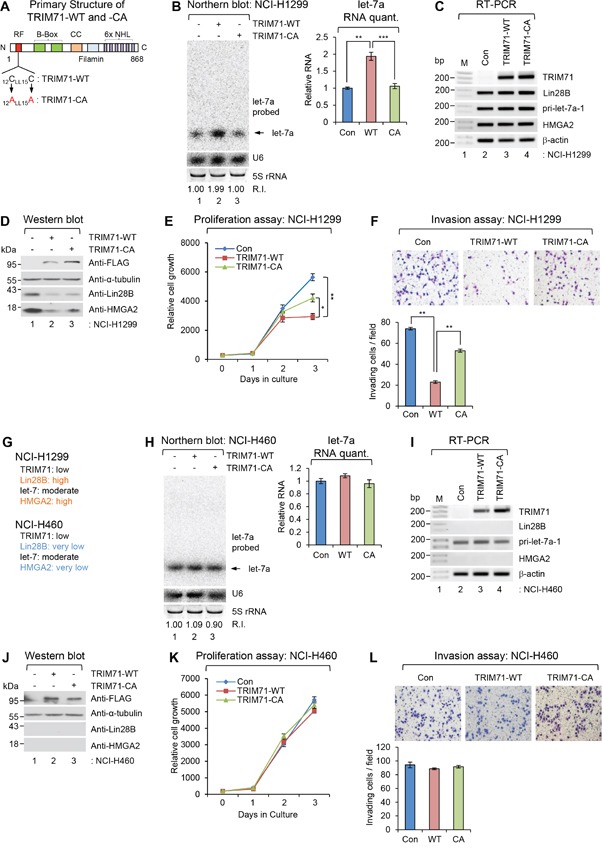
Overexpression of wild-type TRIM71, but not its RING finger mutant, abrogates proliferation and invasion of NCI-H1299 NSCLC cells **A.** Primary Structure of wild type TRIM71 and its RING finger mutant. Two essential cysteine residues located N-terminal RING finger motif were mutagenized as alanine residues (CA). **B-D.** Non-small cell lung cancer cell (NSCLC) NCI-H1299 was infected with control (Con), FLAG-TRIM71(WT), or its RING finger mutant [FLAG-TRIM71(CA)] expressing lentiviral construct. (B) The level of endogenous mature let-7a was monitored by NB with let-7a-specific [^32^P] 5′-end-labelled oligonucleotide probe. Arrow indicates position of mature let-7a. 5S rRNA was used as the loading control and U6 was used as hybridization control (lower left). The quantification of mature let-7a is shown in graph (right). The experiments were repeated at least three times with similar results. The images shown in panel are representative. **p<0.01 and ***p<0.001. (C) The level of TRIM71, Lin28B, HMGA2, β-actin mRNAs, and pri-let-7a-1 RNA were analyzed with RT-PCR in NCI-H1299 cells. M, 100bp ladder. (D) The protein level of FLAG-TRIM71s, α-tubulin, Lin28B, and HMGA2 were analyzed with WB in NCI-H1299 cells. **E.** Proliferation assay was performed in NCI-H1299 cells infected with control (Con), FLAG-TRIM71(WT), or its RING finger mutant [FLAG-TRIM71(CA)] expressing lentiviral construct. *p<0.05 and **p<0.01. **F.** Invasion assay was performed in NCI-H1299 cells infected with control (Con), FLAG-TRIM71(WT), or its RING finger mutant [FLAG-TRIM71(CA)] expressing lentiviral construct. Representative photos of invaded cells (upper) and quantitative data of invasion cells (lower) were shown. **p<0.01. **G.** Gene expression profiles of TRIM71, Lin28B, let-7, and HMGA2 in NSCLC NCI-H1299 and NCI-460. **H.** The level of endogenous mature let-7a was monitored by NB in NSCLC NCI-H460 cells infected with control (Con), FLAG-TRIM71(WT), or its RING finger mutant [FLAG-TRIM71(CA)] expressing lentiviral construct. **I.** The level of TRIM71, Lin28B, HMGA2, β-actin mRNAs, and pri-let-7a-1 RNA were analyzed with RT-PCR in NCI-H460 cells. **J.** The protein level of FLAG-TRIM71s, α-tubulin, Lin28B, and HMGA2 were analyzed with WB in NCI-H460 cells. **K.** Proliferation assay was performed in NCI-H1299 cells infected with control (Con), FLAG-TRIM71(WT), or its RING finger mutant [FLAG-TRIM71(CA)] expressing lentiviral construct. **L.** Proliferation assay was performed in NCI-H460 cells infected with control (Con), FLAG-TRIM71(WT), or its RING finger mutant [FLAG-TRIM71(CA)] expressing lentiviral construct.

To test this hypothesis, we overexpressed wild type TRIM71 (TRIM71-WT) and its RING finger mutant (TRIM71-CA) in the NSCLC cell line NCI-H1299, which expresses high levels of Lin28B and HMGA2, and in the NCI-H460 cell line, which does not express either protein, using lentivirus transduction. Notably, little or no endogenous TRIM71 was detectible in either NSCLC cell line. Lin28B- and HMGA2-high NCI-H1299 cells overexpressing TRIM71-WT showed increased let-7 microRNA biogenesis and reduced Lin28B and HMGA2 protein expression (Figure 4B-4D). NCI-H1299 cell proliferation and invasion were also significantly decreased by overexpression of TRIM71-WT (Figure 4E and 4F). Overexpression of TRIM71-CA in NCI-H1299 cells also partially inhibited proliferation and invasion, possibly owing to potential inhibitory effects of unidentified TRIM71 motifs on tumorigenic phenotypes (Figure 4F).

By comparison, overexpression of TRIM71-WT or TRIM71-CA had no effect on let-7 RNA or tumorigenic phenotypes in Lin28- and HMGA2-non-expressing NCI-H460 cells (Figure 4G-4L). These results indicate that the RING finger motif in the N-terminal region of TRIM71 acts as a key mediator of Lin28B-let-7-HMGA2 signaling. Moreover, disruption of this RING finger motif resulted in abrogation of proliferation and invasion of cancer cells in which Lin28B-let-7-HMGA2 signaling is conserved.

### Tumorigenesis-inhibitory function of TRIM71 is antagonized by overexpression of its downstream targets, Lin28B and HMGA2, in NCI-H1299 cells

Since Lin28B and HMGA2 are downstream post-transcriptional targets of TRIM71, we co-overexpressed Lin28B or HMGA2 with TRIM71 to determine whether Lin28B or HMGA2 overexpression overcomes the inhibitory effect of TRIM71 on tumorigenic phenotypes (proliferation and invasion) of cancer cells.

**Figure 5:**
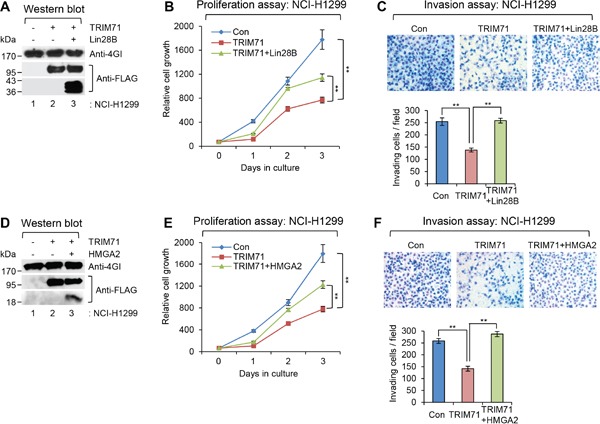
Tumorigenesis-inhibitory function of TRIM71 is antagonized by overexpression of its downstream targets, Lin28B and HMGA2, in NCI-H1299 cells **A.** WB was performed in NCI-H1299 cells infected with control (Con), FLAG-TRIM71(WT), or FLAG-TRIM71(WT) + FLAG-Lin28B(WT) expressing lentiviral construct. The protein level of eIF4GI, FLAG-TRIM71(WT), and FLAG-Lin28B were analyzed. **B.** Proliferation assay was performed in NCI-H1299 cells infected with control (Con), FLAG-TRIM71(WT), or FLAG-TRIM71(WT) + FLAG-Lin28B(WT) expressing lentiviral construct. **p<0.01. **C.** Invasion assay was performed in NCI-H1299 cells infected with control (Con), FLAG-TRIM71(WT), or FLAG-TRIM71(WT) + FLAG-Lin28B(WT) expressing lentiviral construct. Representative photos of invaded cells (upper) and quantitative data of invasion cells (lower) were shown. **p<0.01. **D.** WB was performed in NCI-H1299 cells infected with control (Con), FLAG-TRIM71(WT), or FLAG-TRIM71(WT) + HMGA2-FLAG expressing lentiviral construct. The protein level of eIF4GI, FLAG-TRIM71(WT), and HMGA2-FLAG were analyzed. **E.** Proliferation assay was performed in NCI-H1299 cells infected with control (Con), FLAG-TRIM71(WT), or FLAG-TRIM71(WT) + HMGA2-FLAG expressing lentiviral construct. **p<0.01. **F.** Invasion assay was performed in NCI-H1299 cells infected with control (Con), FLAG-TRIM71(WT), or FLAG-TRIM71(WT) + HMGA2-FLAG expressing lentiviral construct. Representative photos of invaded cells (upper) and quantitative data of invasion cells (lower) were shown. **p<0.01.

As shown in Figure 5B and 5C, co-expression of Lin28B restored cell proliferation and invasion activity, which were inhibited by overexpression of TRIM71-WT alone in NCI-H1299 cells, respectively. Similar results were observed following co-expression of HMGA2 with TRIM71-WT. As shown in Figure 5E and 5F, co-expression of HMGA2 also restored cell proliferation and invasion, which were inhibited by overexpression of TRIM71-WT alone. Taken together, these data strongly suggest that TRIM71 negatively regulates cell proliferation and invasion in a manner that depends on expression of its downstream targets, Lin28B and HMGA2, in NCI-H1299 cells.

### TRIM71 expression is downregulated in various cancer patient tissues

**Figure 6:**
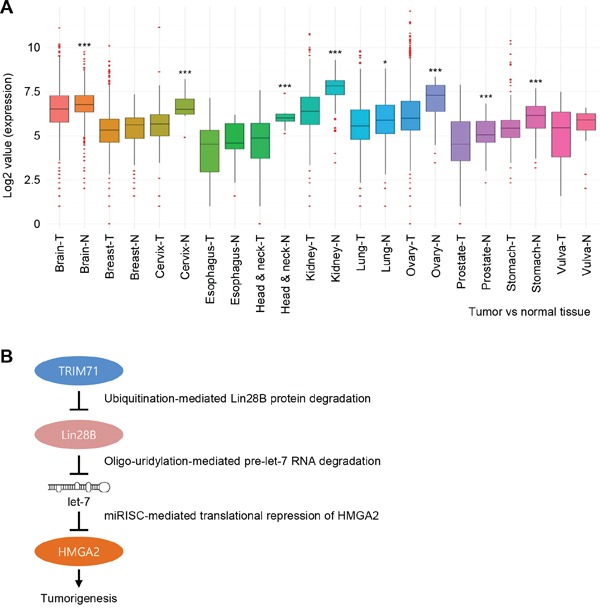
TRIM71 expression is downregulated in various cancer patient tissues **A.** The expression level of TRIM71 obtained from Tumor (T) and Normal (N) in various cancers, including brain, breast, cervix, esophagus, head and neck, kidney, lung, ovary, prostate, stomach, and vulva cancers. TRIM71 gene expression in various cancer patient tissues was obtained from GENT (Gene Expression across Normal and Tumor tissue) database. The data was downloaded to normalized log2 value of TRIM71 gene in the database and the graph was re-drawn in R program. *p<0.05 and ***p<0.001. **B.** Proposed model of TRIM71-mediated modulation of Lin28B, let-7, and let-7 target gene HMGA2 during tumorigenesis. miRISC, miRNA-mediated silencing complex.

Data presented here and shown previously by others indicate that TRIM71 potentially acts as a suppressor of tumorigenesis through regulation of Lin28B-let-7-HMGA2 signaling. To investigate the role of TRIM71 in human cancer patients, we performed a bioinformatics analysis on the GENT (Gene Expression across Normal and Tumor) online tissue database (http://medicalgenome.kribb.re.kr/GENT/). As shown in Figure 6, TRIM71 expression was decreased in cancer tissue compared with normal tissue counterparts, including cancer tissues from the brain, breast, cervix, esophagus, head and neck, kidney, lung, ovary, prostate, stomach, and vulva. Notably, TRIM71 mRNA levels were significantly lower in tumor samples of cervical, head and neck, ovary, stomach, and kidney origin. These results suggest that downregulation of TRIM71 expression is strongly associated with the malignancy of cancers.

As presented in Figure 6B as a plausible model, based on previous and current our findings, we can speculate that strong TRIM71 activity may suppress Lin28B-let-7-HMGA2 linked cellular transformation, since TRIM71 attenuates Lin28B function, thereby inhibiting growth of tumor cells via blockage of oncogenes such as HMGA2 as well as Myc and Ras targeted by let-7.

## DISCUSSION

In the current study, we investigated the post-transcriptional mechanistic interplay of TRIM71 with its downstream target Lin28B and subsequent modulation of let-7-HMGA2 signaling in the regulation of tumorigenesis. TRIM71 is the most-upstream post-transcriptional modulator in the Lin28B-let-7-HMGA2 oncogenic signaling pathway, and serves a pivotal role in tumor formation, tumorigenic progression, and malignancy of cancer. Therefore, we proposed that TRIM71 acts through fine modulation of downstream Lin28B-let-7-HMGA2 signaling to function as a tumorigenic suppressor.

In the current study, we first demonstrated a role for TRIM71 in the transformation process by co-expressing TRIM71 and Lin28B - two main players in the signaling pathway. As shown in Figure 1, TRIM71 completely suppressed Lin28B-mediated transformation of primary mouse embryonic fibroblast NIH/3T3 cells *in vitro*. This observation was confirmed by *in vivo* tumorigenicity assays in which the co-expressing cells were xenografted in nude mice (Figure 2).

Second, we extended this analysis beyond primary cells, introducing siRNA specific for TRIM71 in the cancer cell lines Caco-2 and Tera-1, which express both TRIM71 and Lin28B as well as all components of the conserved signaling pathway. Depletion of TRIM71 in these cancer cell lines clearly recapitulated the results obtained in our *in vitro* transformation assays as well as our *in vivo* tumorigenicity assays. The increase in proliferation and invasion induced by depletion of TRIM71 demonstrated that TRIM71 has a potential tumor-suppressive role. Moreover, an analysis of transcript levels demonstrated that modulation of downstream signaling by TRIM71 is primarily a post-transcriptional event (Figures 1, 3 and 4).

Third, using a gain-of-function strategy, we confirmed the role of the TRIM71 protein itself and intrinsic TRIM71 ubiquitin ligase activity in tumorigenesis in the NSCLC cell lines NCI-H1299 and NCI-H460, respectively. Since the expression level of TRIM71, but not that of other signaling components, is limiting in NCI-H1299 cells, overexpression of TRIM71-WT alone recapitulated the anti-tumorigenic phenotype in these cells. However, in the NSCLC cell line NCI-H460, which is devoid of these signaling components, neither the TRIM71-WT nor TRIM71 ubiquitin transfer-deficient mutant produced a phenotypic response following overexpression. Our results strongly suggest that the RING finger motif is essential for the anti-tumorigenesis function of TRIM71 in addition to its role as a mediator of poly-ubiquitination and protein-protein interactions (Figure 4). Our previous report demonstrated that abolishing the zinc-binding ability of the TRIM71 RING finger by introducing specific point mutations resulted in a loss of ability to interact with Lin28B [6].

In the current study, results obtained using the ubiquitin ligase activity-deficient TRIM71 (TRIM71-CA) suggest that suppression of tumorigenic phenotypes is primarily mediated by the intrinsic ubiquitin-transfer function of TRIM71, and not by other unknown functional motifs in this protein (Figure 4). Collectively, these data suggest that the integrity of the Lin28B-let-7-HMGA2 signaling pathway is critical for TRIM71-mediated tumorigenic suppression.

Finally, the anti-tumorigenic phenotypes driven by TRIM71 were reversed by overexpression of the downstream targets of TRIM71, Lin28B and HMGA2 (Figure 5). Moreover, we found that TRIM71 expression is severely downregulated in tumor tissues from various cancer patients (Figure 6). These data suggest that TRIM71 confers an anti-tumorigenic function in cancer patients in which the Lin28B-let-7-HMGA2 signaling pathway is conserved. Moreover, our data imply that a disruption or imbalance in components of the TRIM71-Lin28B-let-7-HMGA2 signaling cascade can lead to cellular transformation and tumorigenic progression.

### Potential role of ubiquitin ligase activity in tumorigenesis

There is considerable evidence linking deregulation of ubiquitin ligases with cancer development and progression, a linkage that reflects the role of ligases in maintaining normal levels of target oncogenic proteins [1, 2, 20–22]. In certain cases, the role of an ubiquitin ligase in tumorigenesis depends on its specific substrate. For example, the E3 ubiquitin ligase NEDD4-1 modulates protein levels of the well-known tumor suppressor PTEN [3, 22]. The ubiquitin ligase MDM2 has a similar oncogenic relationship with the tumor suppressor p53 [21]. Importantly, our study indicates that disruption of TRIM71 function potentiates tumorigenesis and that over-activation of TRIM71 downregulates Lin28B function as well as downstream signaling. Thus, the relationship between TRIM71 and Lin28B is similar to that between Mdm2 and p53, in which the oncoprotein MDM2 is an E3 ligase for the tumor suppressor p53. Interestingly, post-translational modifications, such as phosphorylation and acetylation, of both MDM2 and p53 regulate the binding and activity of MDM2 toward p53. Similar modes of regulation might also exist for TRIM71 and Lin28B. For example, Lin28B phosphorylation might modulate its ubiquitination by TRIM71. Moreover, TRIM71 possesses multiple domain structures, suggesting that its enzymatic activity could also be closely regulated.

### Substrate specificity of TRIM71 E3 ubiquitin ligase activity

The substrate specificity of the TRIM71 E3 ubiquitin ligase has been reported by several groups [4–6]. Importantly, Lin28A, a close paralog of Lin28B, is not ubiquitinated by TRIM71 [6]. Interestingly, Lin28B and Lin28A critically differ in the C-terminal region at the protein structure level. A lysine- and serine-rich 50-amino-acid stretch makes Lin28B unique in terms of post-translational modulation compared with its paralog Lin28A [6]. Setting stemness considerations aside and focusing at the tumorigenesis level, the relationship of TRIM71 with Lin28B is closer and more critical than that with Lin28A. Moreover, evolutionary forces have shaped the specificity of Lin28B as an ubiquitination substrate of TRIM71 involved in cell proliferation as well as other pivotal biological processes, as evidenced by the fact that there is no obvious ortholog of Lin28B in invertebrates, such as worms [13]. Thus, Lin28B may be uniquely controlled by TRIM71 in the vertebrate system through specialized post-transcriptional regulatory mechanisms that do not exist in invertebrate systems.

### Tumor-suppressive role of the TRIM-NHL protein family

As members the TRIM-NHL protein family, TRIM2, TRIM3, TRIM32 and TRIM71 share structural homology in the C-terminus, which contains a filamin domain and NHL-repeat motifs [1, 23–26]. Moreover, TRIM71 shares functional homology with other TRIM-NHL family proteins in terms of ubiquitin ligase activity.

TRIM3 induces growth arrest in cells, an ability that fundamentally depends on its RING-dependent E3 ligase activity [1, 24]. In addition, several groups have implicated TRIM32 in tumor formation based on studies initially performed in *Drosophila melanogaster* [1]. These early studies showed that mutations in brat and mei-26, ortholog of TRIM32 in the fly, cause tumor formation in *Drosophila* [23]. In mammalian cells, TRIM32 has been shown to modulate TNF-mediated apoptosis through ubiquitination of XIAP (X-linked inhibitor of apoptosis) [27]. The resulting degradation of XIAP causes a loss in its inhibitory activity towards pro-apoptotic caspases. These findings suggest a potential tumor-suppressor role for TRIM32.

It should be noted that these TRIM-NHL proteins function as tumor suppressor in various models of cancers [1, 20]. However, TRIM71 is unusual in that, unlike other cancer-related TRIM-NHL protein family, it has not previously been implicated as a tumor suppressor. According to recent reports, TRIM71 also binds to different RNAs and unique molecular substrates [2, 28, 29]. Thus, TRIM71 and other TRIM-NHL proteins may share unexpected functions, such as RNA-binding and -modulating activities.

### Modulation of Lin28B-let-7-HMGA2 signaling by TRIM71 during tumorigenesis

Lin28B, a specific substrate of TRIM71, has been reported to function as an oncogenic protein that promotes *in vitro* transformation of primary cells and tumorigenesis in various models of neuroblastoma, breast epithelial, and intestinal epithelial cancer [12–18]. Upregulation of Lin28B has also been observed in various tumor types, particularly at the initial stage of the disease. Transcriptional activation of Lin28B is closely associated with interactions of the Myc transcription factor with E-boxes in the *Lin28B* promoter in tumor models [30]. Moreover, some reports have shown that transcriptional activation of Lin28B is closely linked to NF-κB binding to a consensus sequence in the first intron of *Lin28B* [31]. Our report clearly shows that, in addition to transcriptional mechanisms linking Lin28B and tumorigenesis, Lin28B oncogenic activity is repressed post-transcriptionally by TRIM71.

The oncogenic function of Lin28B is primarily attributable to its repression of let-7 biogenesis, reflecting the fact that let-7 miRNAs exert their tumor-suppressive functions by negatively regulating the expression of essential oncogenes, such as HMGA2, Myc and Ras, which modulate cell proliferation [8, 9, 11, 32]. However, Lin28B is also reported to have other non-let-7 miRNA functions. One of recent report has shown that Lin28B promotes migration, invasion, and transformation by associating and repressing LGR5 and PROM1 mRNAs that are essential for colon cancer progression [33].

One of these essential let-7 post-transcriptional targets, the oncoprotein HMGA2, belongs to the non-histone chromosomal high-mobility group protein family. Its mRNA contains eight let-7 binding sites in the 3′-UTR, and the corresponding protein contains structural DNA-binding domains consistent with a transcription-regulating function [9, 10]. HMGA2 expression is usually elevated in tumors of colorectal and lung cancer patients in association with decreased survival. Notably, HMGA2 is highly expressed in metastatic NSCLC [34], consistent with its role in promoting tumor metastasis. However, the precise mechanism by which HMGA2 protein expression is regulated and how HMGA2 contributes to tumorigenesis remain to be elucidated.

As shown in the Figure 4, elevated expression of let-7 repressed translation of HMGA2 mRNA transcripts and thereby inhibits NCI-H1299 cell growth, a response that was rescued by expression of an HMGA2 open reading frame without a 3′-UTR (Figure 5). Our current findings, together with previous studies, show that HMGA2 is essential for promoting NSCLC malignancy and metastasis. However, this regulatory mechanism is critically dependent on Lin28B protein, let-7 microRNA, and the presence of authentic let-7 targeting sites in the 3′-UTR region of HMGA2 mRNA. Thus, strong TRIM71 activity potentially suppresses cellular transformation driven by Lin28B-let-7 in tumors in which this pathway is conserved by attenuating Lin28B protein function, thereby inhibiting tumor cell growth through inhibition of potential let-7 target oncogenes, especially HMGA2.

### Functional complexity of TRIM71 in post-transcriptional control

In the current report, we clearly demonstrated a role for TRIM71 during tumorigenesis at the molecular level, providing important new information about the function of the TRIM71 protein. However, additional molecular functions of TRIM71 have remained largely unknown. Several groups have suggested that essential known domains other than the RING finger domain, such as NHL repeats, show RNA-binding activity [2, 28]. Furthermore, related with the RNA-binding, functional interaction between TRIM71 and Ago2 in conjunction with embryonic stem cell-specific miRNAs has shown to post-transcriptionally repress expression of the cell cycle regulator, Cdkn1a [35]. Most recently, Worringer and colleagues showed that TRIM71 can block reprogramming via association with EGR1 mRNA and negatively regulate its translation [36].

Specifically, it has been shown that the NHL domain, one of the defining features of TRIM71, is required for mRNA targeting by TRIM71 and subsequent repression of translation, whereas the ubiquitin ligase activity of the RING finger domain is not [28]. Similarly, we also confirmed that the RING finger motif is indispensable for downregulation of Lin28B protein; however, experiments employing synthetic constructs of Lin28B and TRIM71 showed that it is not involved in translational repression of Lin28B mRNA [6] (data not shown). Therefore, these two domains of TRIM71 are functionally distinct in terms of post-transcriptional modulation of gene expression.

In conclusion, the present study unequivocally demonstrates a novel tumor-suppressive role for the TRIM-NHL protein TRIM71, showing that silencing or targeted depletion of TRIM71 induces activation of the essential LIN28B-let-7-HMGA2 signaling, resulting in increased tumorigenesis *in vitro* and *in vivo*. Taken together with previous reports, our findings shed further light on the roles of TRIM71 in various biological processes, especially tumorigenesis.

## MATERIALS AND METHODS

### Cell culture

293FT, Tera-1, and NIH/3T3 cells were cultured in Dulbecco's modified Eagle medium (DMEM; HyClone). NCI-H1299, NCI-H460, and Caco-2 cells were cultured in RPMI-1640 medium (HyClone). All media were supplemented with 10% fetal bovine serum (HyClone), penicillin/streptomycin and 10 mg/ml ciprofloxacin.

### Plasmids

For the generation of retroviral construct pMSCV-neo-TRIM71, T-Kozak-TRIM71(WT) was digested with EcoRI-XmaI and XmaI-SalI, respectively. Two fragments were inserted into pMSCV-neo (Clontech) which treated with EcoRI-XhoI-CIP. For the generation of pBABE-puro-Lin28B, PCR was performed with specific oligomers (Supplementary Table S1) with pcDNA3-FLAG-Lin28B [6] as a template. BamHI-SalI digested PCR product inserted into pBABE-puro. For the generation of pBABE-puro-KRAS(G12V), PCR was performed with the template pLT-puro-KRAS(G12V). PCR product digested with BamHI-SalI and then inserted into pBABE-puro. For the generation of lentiviral constructs pLenti6-TRIM71(WT) and pLenti6-TRIM71(CA), PCR was performed with pcDNA3-FLAG-TRIM71(WT) and pcDNA3-FLAG-TRIM71(CA) [6] as the templates. The amplified DNA fragments were inserted into EcoRI-XhoI-CIP treated pLenti6-MCS via In-Fusion reaction (Takara), respectively. For the generation of pcDNA3-HMGA2-FLAG, PCR was performed with cDNA from HepG2 cell total RNA as a template. The amplified DNA fragment was inserted into BamHI-XbaI-CIP treated pcDNA-cFLAG. pLenti6-HMGA2-FLAG was generated by In-Fusion reaction with EcoRI-XhoI treated pLenti6-MCS and PCR product of pcDNA3-HMGA2-FLAG. pLenti6-FLAG-Lin28B was constructed by In-Fusion reaction with pcDNA3-FLAG-Lin28B as a PCR template. All oligomers were purchased from Macrogen (Korea). All constructs were verified by DNA sequencing.

### Antibodies and western blotting

Anti-hLin41 (R&D), anti-Lin28B (Cell Signaling Technology), anti-Lin28A (Abcam), anti-β-actin (clone C4, Millipore), anti-α-tubulin (clone TU-02, Santa Cruz Biotech), anti-CPSF73 (Bethyl Laboratories), anti-symplekin (BD Biosciences), anti-HuR (Santa Cruz Biotech), anti-Ras (Abcam), anti-eIF4GI [6], anti-FLAG (clone L5, BioLegend), and anti-HMGA2 (GeneTex) antibodies were used through the all western blot analysis.

### Semi-quantitative RT-PCR

Total RNAs were isolated using TRIzol (Invitrogen) according to the manufacturer's instructions. cDNAs were synthesized using GoScript™ Reverse Transcription System (Promega) with oligo(dT)_20_ and 1 μg of total RNA according to the manufacturer's directions. PCR reaction parameter for each molecule is as followed; 95°C for 20 sec, 55°C for 40 sec, and 72°C for 20 sec. Total number of cycles is as followed; 35 cycles for TRIM71, HMGA2, pri-let-7a-1; 30 cycles for Lin28B, pri-miR-21, and pri-miR-29a; 27 cycles for β-actin. 1 μl of cDNA was used as PCR template for all reaction except β-actin which 1/10 diluted cDNA was used as a template. The PCRs were performed with e-Taq (Solgent) and specific oligomers (Supplementary Table S2) (Macrogen). The PCR products were analyzed on the 1% agarose gel.

### Lentivirus production and infection

Lentivirus production, infection, proliferation, and invasion assay were performed as described previously [37, 38, 39]. Retroviruses were generated from two retroviral systems, pBABE-puro and pMSCV-neo. pBABE-puro, pBABE-puro-Lin28B, pBABE-puro-KRAS(G12V), pMSCV-neo, and pMSCV-neo-TRIM71 plasmids were cotransfected with pCMV-VSV-G and pCMV-gag-pol into 293T cells. Retroviral supernatants were collected at 48 hr after transfection. 24 hr before infection, 2 × 10^5^ NIH/3T3 cells were plated on 35 mm culture dishes. Two step sequential infections were used for the generation of each combination of expression. Double-infected NIH/3T3 cells were selected with 2 μg/ml of puromycin and 400 μg/ml of G418 together for one week more. Stable NIH/3T3 cell lines were suspended in 0.35% agarose with DMEM containing 10% FBS. 5,000 cells/well of retrovirus infected stable NIH/3T3 cells were plated on 6-well plates for soft agar colony formation assay. The plates were placed in an incubator around 4 weeks.

### Tumorigenicity and immunofluorescence assay

Five weeks old female BALB/c nude mice were purchased from Charles River Inc. (Japan). Mice adapt to the new surroundings one week before to inject the retrovirus infected cells. For the tumorigenicity experiment, 5 × 10^6^ of retrovirus-infected NIH/3T3 cells in 150 μl of 1× DPBS were subcutaneously injected into the hip area on both sides of each mouse. Tumor growth was measured three times a week using electronic caliper to measure two diameters by the formula: length × width^2^ × 0.5. The mean tumor volume at the start of the experiment was 23 mm^3^. Tumors were grown until they reached a median size of ~1,000 mm^3^ (4~5 weeks). Mice were sacrificed 5 weeks after retrovirus infected NIH/3T3 cells injections. The tumors were extracted, pooled for each experimental group, and mechanically disaggregated through a steel operating scissors. All animal studies were approved by the Institutional Animal Care and Use Committee (IACUC) review board of National Cancer Center and conducted under the guidelines of the National Cancer Center IACUC. Immunofluorescence assay was performed as described previously [37]. The following antibodies were used: anti-Ki-67 (1:1,000; Novocastra), and anti-HMGA2 (1:100).

### Small RNA analysis by northern blotting and splinted ligation

Northern blot (NB) and splinted ligation were performed as described previously [6, 38]. In brief, total RNAs were isolated with TRIzol and NB was carried out *N*-(3-Dimethylaminopropyl)-*N'*-ethylcarbodiimide (EDC; Sigma-Aldrich) cross-linking method. Sequence of oligonucleotides are shown in Supplementary Table S3.

### Statistical analysis of data

Data are presented as the mean ± standard deviation (SD) determined from minimum three independent experiments. Differences were assessed by the two-tailed Student's t-test using Excel software (Microsoft). *p*≤ 0.05 was considered as statistically significant.

## SUPPLEMENTARY FIGURE AND TABLES


