# Supplementary material for: TRIM71 suppresses tumorigenesis via modulation of Lin28B-let-7-HMGA2 signaling
Source: Oncotarget. 2016 Nov 3;7(48):79854–68. doi: 10.18632/oncotarget.13036 (PMC5346756; doi:10.18632/oncotarget.13036)
Supplement: Supplementary file 1 [file oncotarget-07-79854-s001.pdf]

TRIM71 suppresses tumorigenesis via modulation of Lin28B-let-7-HMGA2 signaling

SUPPLEMENTARY FIGURE AND TABLES

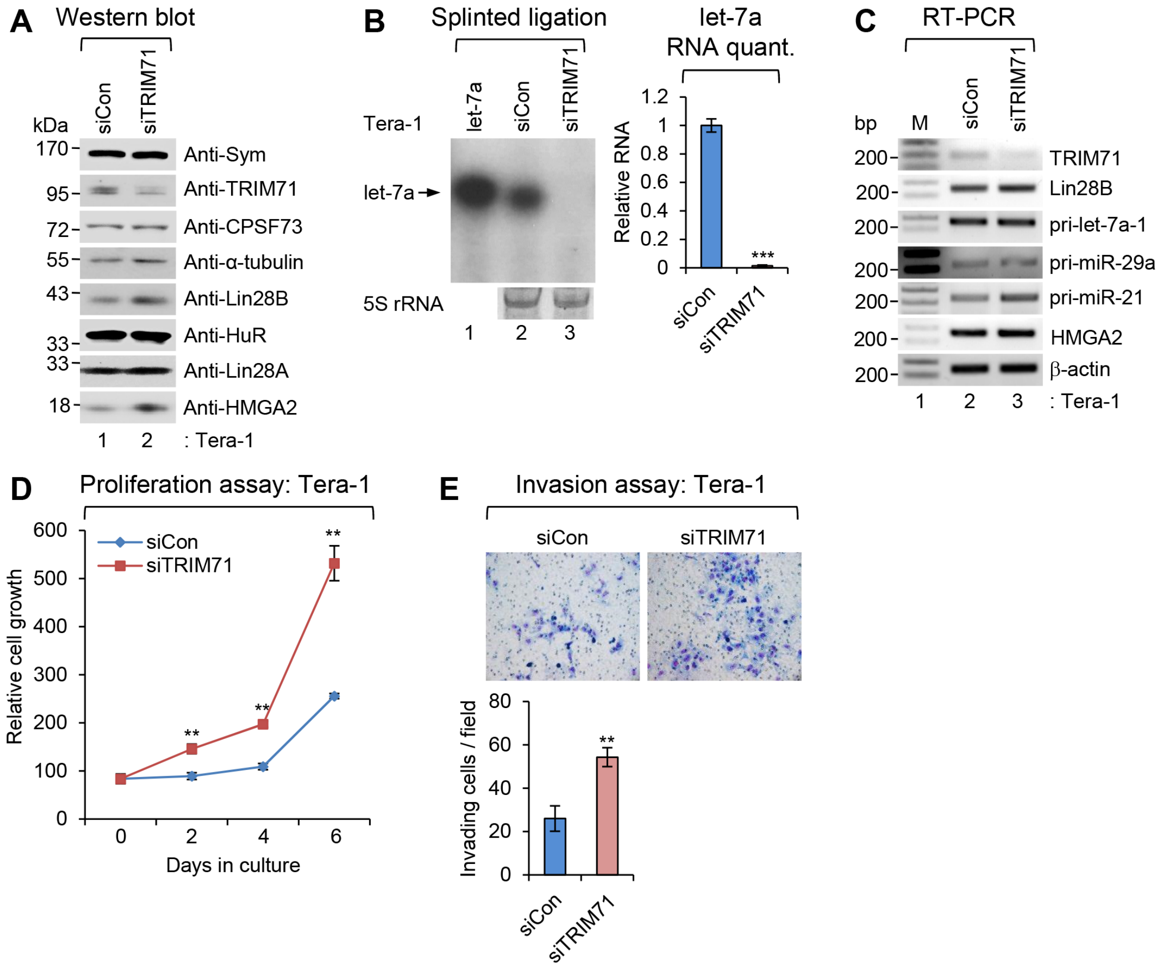

Supplementary Figure S1: Depletion of TRIM71 promotes proliferation and invasion of human embryonal carcinoma Tera-1 cells. **A.** The level of symplekin, TRIM71, CPSF73, α-tubulin, Lin28B, HuR, Lin28A, and HMGA2 proteins were confirmed by WB after specific knockdown of TRIM71 in human embryonal carcinoma Tera-1 cells. **B.** Splinted ligation was performed with [^{32}P] 5′-end-labeled oligonucleotide probe specific for mature let-7a as described in MATERIALS AND METHODS. Aliquot of total RNAs were resolved in 12% SequaGel together as the loading control (5S rRNA). The experiments were repeated at least three times with similar results. The images shown in panels are representative one. ***p<0.001. **C.** The level of TRIM71, Lin28B, HMGA2, β-actin mRNAs, and various pri-miRNAs were analyzed with RT-PCR. **D.** Proliferation assay was performed in Tera-1 cells transfected with scrambled (siCon) or TRIM71 (siTRIM71) specific siRNA. **p<0.01. **E.** Invasion assay was performed in Tera-1 cells transfected with scrambled (siCon) or TRIM71 (siTRIM71) specific siRNA. Representative photos (top) and quantitative data of invaded cells (lower) are shown. **p<0.01.

Supplementary Table S1: Oligonucleotides used in PCR-based cloning of various plasmid constructs

Plasmid constructs	Sequence of oligonucleotides
pBABE-puro-Lin28B	S: 5′-CGGGATCCGCCACCATGGCCGAAGGCGGGGCTAG-3′<br>A: 5′-ACGGTCGACTTATGTCTTTTTCCTTTTTTGAAC-3′
pBABE-puro-KRAS(G12V)	S: 5′-CGGGATCCGCCACCATGACTGAATATAAACTTG-3′<br>A: 5′-CCGCTCGAGTTACATAATTACACACTTTG-3′
pLenti6-TRIM71(WT) & pLenti6-TRIM71(CA)	S: 5′-AGTGTGGTGGAATTCGCCACCATGGACTACAAG-3'<br>A: 5′-CCCTCTAGACTCGAGGATTAGAAGACGAGGATTCG-3'
pcDNA3-HMGA2-FLAG	S: 5′-CGGGATCCGCCACCATGAGCGCACGCGGTGAGGGC-3'<br>A: 5′-GCTCTAGACCGTCCTCTTCGGCAGACTCTTGTGAG-3'
pLenti6-HMGA2-FLAG	S: 5′-AGTGTGGTGGAATTCGGATCCGCCACCATGAGCG-3'<br>A: 5′-CCCTCTAGACTCGAGGGCCCTACTTGTCATCGTCG-3'
pLenti6-FLAG-Lin28B	S: 5′-AGTGTGGTGGAATTCGCCACCATGGACTACAAG-3'<br>A: 5′-CCCTCTAGACTCGAGTAAGCTTGGTACCTTATGTC-3'

* S, sense; A, antisense.

Supplementary Table S2: Oligonucleotides used in semi-quantitative RT-PCR analysis

Genes	Sequence of oligonucleotides
TRIM71	S: 5′-CACGACGACGAGGTGCTGCAC-3′<br>A: 5′-GGGCCTGCTCGATGCTCAGC-3′
Lin28B	S: 5′-GCAAAGGTGGTGGAGAAGAG-3′<br>A: 5′-GGCTTCCCTCTCGGTTTATC-3′
pri-miR-21	S: 5′-CGGGATCCAAATCCTGCCTGACTGTCTGC-3′<br>A: 5′-GGAATTCTGATTATAAACAATGATGCTGG-3′
pri-miR-29a	S: 5′-CGGGATCCAAGAGCCCAATGTATGCTGG-3′<br>A: 5′-GGAATTCAACGGTCACCAATACATTTCC-3′
pri-let-7a-1	S: 5′-GATTCCTTTTCACCATTCACCCTGGATGTT-3′<br>A: 5′-TTTCTATCAGACCGCCTGGATGCAGACTTT-3′
β-actin	S: 5′-CCTGGAACGGTGAAGGTGACA-3′<br>A: 5′-AAGGGACTTCCTGTAACAATGCA-3′
Mouse β-actin	S: 5′-CTGTCCCTGTATGCCTCTG-3′<br>A: 5′-ATGTCACGCACGATTTCC-3′
HMGA2	S: 5′-CAGCAGCAAGAACCAACCG-3′<br>A: 5′-GTTGTGGCCATTTCCTAGGT-3′
Mouse HMGA2	S: 5′-AAGGCAGCAAAAACAAGAGC-3′<br>A: 5′-AATCCTCCTCTGCGGACTCT-3′

* S, sense; A, antisense.

Supplementary Table S3: Oligonucleotides used in northern blotting, splinted ligation, and knock down experiment for TRIM71

Name	Sequence of oligonucleotides
anti-let-7a	5′-AACTATACAACCTACTACCTCA-3′
anti-U6	5′-GCTTCACGAATTTGCGTGTCATCCT-3′
let-7a RNA	5′-UGAGGUAGUAGGUUGUAUAGUU-3′
let-7a bridge oligonucleotide	5′-GAATGTCATAAGCGAACTATACAACCTACTACCTCA-3′
ligation oligonucleotide	5′-CGCTTATGACATTC/dideoxyC/-3′
siCon	5′-GUUCAGCGUGUCCGGCGAG-3′
siTRIM71	5′-CCUGUGCAAGUUUGGUGCU-3′
